# Evaluating ChatGPT-4o as an Educational Support Tool for the Emergency Management of Dental Trauma: Randomized Controlled Study Among Students

**DOI:** 10.2196/80576

**Published:** 2025-11-20

**Authors:** Franziska Haupt, Tina Rödig, Paula Liersch

**Affiliations:** 1 Department of Preventive Dentistry, Periodontology and Cariology University Medical Center Göttingen Göttingen, Lower Saxony Germany

**Keywords:** decision–making support tool, dental trauma education, large language model, mobile app, prompting strategy

## Abstract

**Background:**

Digital tools are increasingly used to support clinical decision-making in dental education. However, the accuracy and efficiency of different support tools, including generative artificial intelligence, in the context of dental trauma management remain underexplored.

**Objective:**

This study aimed to evaluate the accuracy of various information sources (chatbot, textbook, mobile app, and no support tool) in conveying clinically relevant educational content related to decision-making in the primary care of traumatically injured teeth. Additionally, the effect of the input strategy on the chatbot’s output response was evaluated.

**Methods:**

Fifty-nine dental students with limited prior experience in dental trauma were randomly assigned to one of 4 groups: chatbot (based on generative pretrained transformer [GPT]-4o, n=15), digital textbook (n=15), mobile app (AcciDent app 3.5, n=15), and control group (no support tool, n=14). Participants answered 25 dichotomous questions in a digital examination format using the information source allocated to their group. The primary outcome measures were the percentage of correct responses and the time required to complete the examination. Additionally, for the group using ChatGPT-4o, the quality of prompts and the clarity of chatbot responses were independently evaluated by 2 calibrated examiners using a 5-point Likert scale. Statistical analyses included nonparametric analyses using Kruskal-Wallis tests and mixed-effects regression analyses with an α level of .05.

**Results:**

All support tools led to a significantly higher accuracy compared with the control group (*P*<.05), with mean accuracies of 87.47% (SD 5.63%), 86.40% (SD 5.19%), and 86.40% (SD 6.38%) for the textbook, the AcciDent app, and ChatGPT-4o, respectively. The groups using the chatbot and the mobile app required significantly less time than the textbook group (*P*<.05). Within the ChatGPT-4o group, higher prompt quality was associated with greater clarity of the chatbot’s responses (odds ratio 1.44, 95% CI 1.13-1.83, *P*<.05), which in turn increased the likelihood of students selecting the correct answers (odds ratio 1.89, 95% CI 1.26-2.80, *P*<.05).

**Conclusions:**

ChatGPT-4o and the AcciDent app can serve dental students as an accurate and time-efficient support tool in dental trauma care. However, the performance of ChatGPT-4o varies with the precision of the input prompt, underscoring the necessity for users to critically evaluate artificial intelligence–generated responses.

**Trial Registration:**

OSF Registries 10.17605/OSF.IO/XW62J; https://osf.io/xw62j/overview

## Introduction

Dental trauma is defined as an acute mechanical injury to teeth and surrounding structures, commonly resulting from accidents or falls. With a global prevalence of 25%-30%, dental trauma represents a significant public health issue, affecting approximately 1 billion individuals and ranking as the fifth most common type of injury worldwide [1,2]. The incidence of dental trauma has shown an upward trend in recent years, leading to considerable health care expenditures [3]. In Germany alone, the estimated annual costs related to dental trauma range between €200 and €550 million (€1=US $1.03) [4].

Immediate and appropriate treatment is crucial to prevent long-term complications and to reduce treatment-related costs [4]. Inadequate or inappropriate initial care is a major contributing factor to poor outcomes following dental trauma. Therefore, timely and accurate diagnosis combined with evidence-based primary treatment is essential to optimizing functional and aesthetic prognoses [5,6]. To standardize the management of dental trauma, several clinical practice guidelines have been published [7]. Among these, the guidelines developed by the International Association of Dental Traumatology (IADT) provide clearly structured recommendations, which, when followed, have been associated with improved clinical outcomes and a reduced incidence of complications [8-10]. Unfortunately, it has been shown that there is substantial evidence of educational shortcomings in the field of dental trauma [11,12], which is further underscored by studies reporting varying, partly insufficient knowledge among dental professionals [13,14].

According to a recent systematic review, mobile health approaches have gained increasing popularity among both patients and health care professionals [15]. Mobile health refers to the emerging field of health care that uses mobile-based technologies, such as smartphone apps, social media platforms, and artificial intelligence (AI), for disease prevention, health education, and clinical decision support [15,16]. To date, several mobile apps have been developed, including 3 based on the IADT guidelines (ToothSoS, AcciDent, and Injured tooth) [15]. Given the growing availability and integration of health care technologies, it appears likely that students tend to compensate for knowledge deficits in dental trauma management by turning to digital solutions [17].

Moreover, AI-driven systems have gained increasing relevance in medical and dental practice in recent years [18-20]. Advances in AI have accelerated the development of large language models (LLMs), such as generative pretrained transformer (GPT) [21], which are increasingly recognized for their potential to assist in medical practice [22]. However, when using chatbots based on LLMs, obtaining high-quality answers largely depends on how users interact with the chatbot [23,24]. For this purpose, the emerging field of prompt engineering aims to systematically develop prompts to enhance the performance of LLMs [23,25]. Nevertheless, awareness among practitioners regarding the importance of effective prompting and the potential impact of various prompting techniques on the quality of generated output remains limited [23].

Previous research in dental traumatology has evaluated the accuracy of chatbot-generated responses in simulated dental trauma scenarios. Ozden et al [26] reported that the performance of such models varied significantly depending on the specific algorithm. In their evaluation, ChatGPT-3.5 demonstrated an accuracy of 51%, whereas Google Bard achieved a slightly higher accuracy of 64% [26]. More recent findings, however, suggest substantial advancements in the performance of newer LLMs. A recently published study assessing the emergency management of tooth avulsion found that ChatGPT-4 attained an accuracy of up to 96.3% in responding to dichotomous (yes/no) clinical questions [27].

Although AI apps in health care are expanding rapidly, further research is necessary to evaluate their reliability and clinical applicability in dentistry [28]. Given their ability to provide rapid, accessible information, LLMs may represent a valuable resource for managing dental trauma in emergency settings. Moreover, they are likely to be used by students as a supplementary tool during exam preparation. Nevertheless, as previously noted, the effectiveness of chatbot responses remains highly dependent on the specificity and clarity of user input. Therefore, the primary objectives of this study were to evaluate the accuracy of 3 different support tools compared with a control group by assessing the percentage of correct responses and to record the total time required. The null hypotheses stated that there would be no significant differences among the 4 groups in terms of response accuracy or time efficiency. As a secondary objective, the study examined whether the precision of the input prompt submitted to the chatbot influenced the clarity of its responses (output), and whether this output clarity affected the students’ likelihood of selecting the correct answer.

## Methods

### Ethical Considerations

The study was approved by the local ethics committee of the University Medical Center Göttingen, Germany (number 28/10/24) and conducted in accordance with the Declaration of Helsinki (Registration on OSF Registries osf.io/xw62j). Dental students in their final academic year were asked to participate in the study. All of them had signed informed consent prior to participation. The researchers had no access to personal information such as sex, age, or prior academic performance. All data were pseudonymized before being subjected to statistical analysis. Participants did not receive any financial compensation. As an incentive, the 5 best-performing students in each group were awarded a book prize.

### Study Design

A consecutive randomization procedure was applied to dental students in their final academic year, comprising participants from the 9th (n=26) and 10th semesters (n=33), resulting in a total sample of 59 students. Selection was conducted in a blinded manner, ordered by ascending matriculation numbers. Students were pseudorandomly allocated into 4 groups, ensuring that each group included an equal number of students from the 9th and 10th semesters. This approach ensured allocation concealment and minimized potential selection bias. None of the students had received formal education in dental traumatology, ensuring a homogenous baseline knowledge across groups. They were not informed about the study content or its design until shortly before the start of the study, in order to prevent individual preparation. At this point, informed consent was required for participation. Based on group allocation, each student was granted access to one specific support tool: control (no tool, n=14), a digital textbook (n=15) [29], a mobile app (AcciDent app 3.5, n=15), or a GPT-4o-based chatbot (OpenAI, n=15). Students using the digital textbook [29] were instructed to use the keyword-based search function. Using a computer-based assessment tool (CAMPUS version 1.4 Rev 7090; IMS-3, Institut für Kommunikations-und Prüfungsforschung gGmbH), all students completed 25 questions presented as declarative statements, which they were instructed to classify as either true or false, using only their assigned support tool (Table S1 in Multimedia Appendix 1).

To ensure standardization and minimize potential bias, all participants in the GPT-4o chatbot group were provided with newly created accounts that had no prior usage history. Each assessment session was initiated with a fresh login and no prior interactions, effectively eliminating session memory effects. Students accessed the chatbot via a browser-based interface under supervised conditions. No other prompts or unrelated queries were entered during the assessment. This setup ensured that all responses were generated based solely on the test items and not influenced by prior context, history, or personalized adaptation of the model.

The 25 dichotomous assessment questions were developed by one specialized endodontist and one general dentist with clinical expertise in endodontics. The questionnaire was based on current IADT guidelines [5,6,9], which provide evidence-based recommendations for dental trauma management.

To eliminate time-related variability within the chatbot group, the assessment was conducted simultaneously for all study participants on December 11, 2024. During the experiment, the total time required to complete the questionnaire was recorded for each student. Participants were informed that their working time would be recorded, but no time limits were imposed in order to prevent influencing their performance. All answers were pseudonymized and transferred to an Excel spreadsheet (Microsoft Corp) for statistical analysis.

### Item Analysis and Scoring Criteria

To ensure content validity, the items were reviewed by 5 dental education experts prior to the experiment. All questions were reviewed to confirm they could be answered using one of the assessed supporting tools. For the item quality analysis, all items were evaluated using the difficulty index (P), the discrimination index (D), and internal consistency reliability (Kuder Richardson formula 20; KR-20). In addition, group-specific difficulty and discrimination rates for the control group were calculated in order to better reflect the discriminatory power of the items in relation to baseline student knowledge. The difficulty index ranges between 0% and 100%, with the latter representing the lowest difficulty. Based on established guidelines in educational measurement [30,31], the ideal difficulty of items should be at a point on the difficulty scale midway between 100% and the chance level difficulty (50% for true-false items). This means the ideal difficulty index (P) value should be about 0.75 for dichotomous questions [31]. The discrimination index is the ability of an item to differentiate between students of higher and lower abilities and ranges between 0 and 1 [32]. The KR-20 reliability coefficient is a measure of reliability for a test with binary variables, ranging from 0 to 1, where 0 is no reliability and 1 is perfect reliability. In general, a score of more than 0.5 is usually considered reasonable.

A prompting strategy analysis was conducted within the chatbot group. For this purpose, all chat transcripts were exported and subsequently stored in pseudonymized form as PDF files. Based on previous publications, 2 independent raters evaluated each interaction using a 5-point Likert scale (1=strongly disagree, 2=disagree, 3=neutral, 4=agree, and 5=strongly agree) [33,34]. In cases of disagreement between the 2 raters, a consensus was achieved by reevaluation. This process ensured the achievement of a unanimous result while maintaining the integrity and objectivity of a dual-rater system. The scale was specifically developed in alignment with established prompting techniques, including role prompting, chain-of-thought prompting, knowledge-guided prompting, and output format specification. The following aspects were assessed: (1) the extent to which the student’s prompt was focused, specific, and aligned with effective prompting strategies (input evaluation), and (2) the clarity and persuasiveness of the chatbot’s response (output evaluation; Table 1). Furthermore, the Flesch Reading Ease was used to assess the readability and complexity of the answers provided by ChatGPT-4o, with results presented as a numerical value ranging from 1 to 100 [35].

**Table 1:** Predefined criteria for evaluating the input prompt and the output response given by the chatbot. For each applicable criterion, one point was assigned, and the corresponding value on the Likert scale was applied.

Evaluated element	Predefined criteria for chat evaluation
Input prompt	Focused question, inclusion of context or domain-specific details, precise requirements for the output (such as format), reasoning, reference to evidence
Output	Content-related, clear and persuasive, with justification, use of specific medical terms, and reference to guidelines

### Statistical Analysis

As the data were not normally distributed, Kruskal-Wallis tests were applied to analyze potential differences between groups in terms of the percentage of correct responses and the total time required. Multiple post hoc pairwise comparisons were performed (using Dunn tests), and the resulting *P* values were adjusted using the Bonferroni-Holm method. To assess the internal agreement within each group, Fleiss κ coefficients were computed [36].

Regarding the prompting strategy, interrater agreement was calculated using weighted Cohen κ. Based on the evaluations of input and output quality, 2 regression analyses were carried out. (1) An ordinal regression model with a random intercept was applied to analyze the effect of the input prompt on the ordinal outcome variable output response. The student was included as a random effect to account for interindividual variability. Confidence intervals were computed using profile likelihood estimation. (2) A logistic regression model with a random intercept for the student was applied to examine the effect of the output response on the students’ likelihood of selecting the correct dichotomous answer. Confidence intervals were estimated using profile likelihood methods.

All statistical analyses were performed using the software R (version 4.4.2; R Foundation for Statistical Computing). The predetermined α level was .05.

## Results

**Figure 1:**
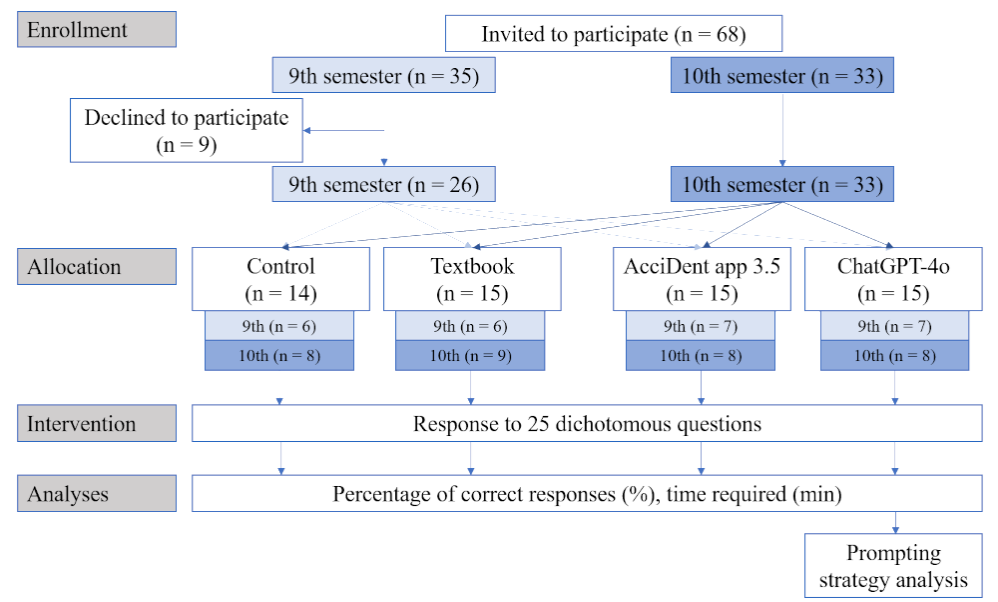
Research design and participant allocation into the groups.

The participant flow diagram is shown in Figure 1. Regarding the item analysis, the KR-20 reliability coefficient was 0.59. The results of the item difficulty index and the discrimination index are summarized in Table 2 and presented in greater detail in Table S2 in Multimedia Appendix 2.

**Table 2:** Mean and SD and median and range of item difficulty and discrimination indices among all groups and for the control group across all 25 items.

	Total			Control group	
	Mean (SD)	Median (range)	Mean (SD)		Median (range)
Difficulty index	0.84 (0.16)	0.92 (0.47 to 1.0)	0.77 (0.25)		0.86 (0.29 to 1.0)
Discrimination index	0.18 (0.18)	0.12 (0.01 to 0.56)	0.20 (0.29)		0.07 (–0.20 to 1.0)

**Figure 2:**
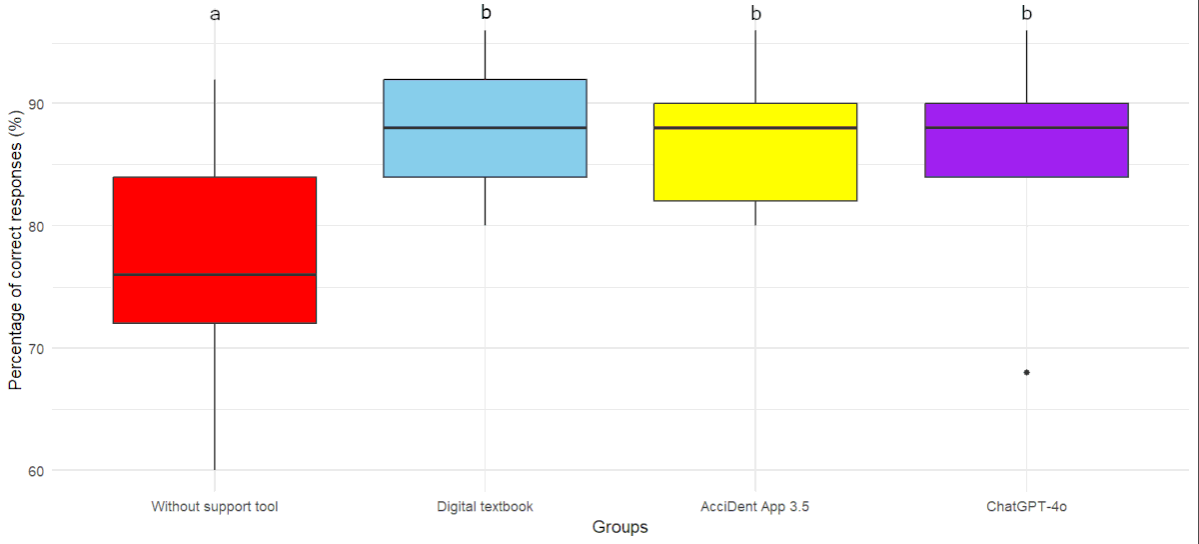
Boxplots illustrating the percentage of correct responses among the 4 experimental groups. Small letters indicate statistically significant differences among groups.

**Figure 3:**
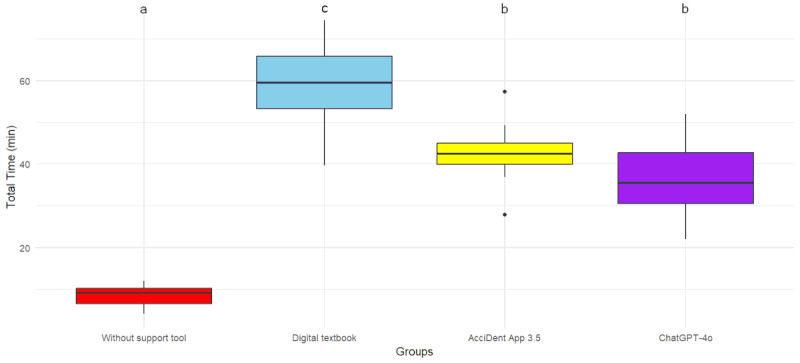
Boxplots illustrating the time required to complete the questionnaire among the 4 experimental groups. Small letters indicate statistically significant differences among groups.

The control group achieved the lowest percentage of correct responses, with statistically significant differences compared with all 3 groups having used an information source (*P*_Textbook_=.006, *P*_App_*=*.02, *P*_Chatbot_*=*.008; Figure 2, Table 3). In terms of time required, the control group demonstrated the fastest performance, when compared with all groups using a support tool (*P*_Textbook_*<*.001, *P*_App_*<*.001, *P*_Chatbot_*=*.005). Of these, the textbook group required the most time on average, with significant differences to the AcciDent app group (*P=.*02) and the chatbot group (*P=*.001). The latter showed no significant difference between each other (*P=.*37). Internal agreement was highest within the chatbot group, followed by the AcciDent app group, both showing moderate agreement. (Figure 3 and Table 3).

**Table 3:** Statistics regarding the percentage of correct answers and the total time required, as well as the Fleiss κ coefficient for the experimental groups. Small letters indicate statistically significant differences among groups.

	Percentage of correct responses			Time required (min)			Fleiss κ
	Mean (SD)	Median (IQR)	Mean (SD)		Median (IQR)		
Control	76.57 (8.71)	76 (72-84)^a^	8.54 (2.45)		9.03 (6.55-10.20)^a^	0.28	
Textbook	87.47 (5.63)	88 (84-92)^b^	59.60 (10.39)		59.50 (53.62-65.90)^c^	0.24	
AcciDent app 3.5	86.40 (5.19)	88 (82-90)^b^	42.65 (6.44)		42.52 (39.89-45.03)^b^	0.41	
ChatGPT-4o	86.40 (6.38)	88 (84-90)^b^	36.56 (10.03)		35.53 (30.58-42.78)^b^	0.47	

^a,b,c^Statistically significant differences among groups.

In the quality analysis, the input prompts posed by the students received a median rating of 2 (IQR 2-3, range 1-5), while the chatbot’s output responses were rated with a median of 4 (IQR 3-4, range 1-5). Cohen κ indicated a high level of interrater agreement, with values of 0.98 and 0.82 for the input prompts and the output responses, respectively.

The ordinal regression analysis revealed a significant effect of the input prompt on the output response with an effect estimate of 0.3647 (*P*=.003). The estimated (odds ratio 1.44, 95% CI 1.13-1.83) suggests that for each unit increase in prompt precision, the odds of being in a higher category for the output response increased by 44%.

The logistic regression model demonstrated a significant effect of the chatbot’s output response on the selection of the correct answer (effect estimate=0.6345, *P*=.002). The odds ratio of 1.89 (95% CI 1.26-2.80) indicates an 89% increase in the odds of correctness for each unit increase in clarity of the output response.

According to the Flesch Reading Ease, the answers given by ChatGPT-4o had a mean value of 36.20 (SD 9.13), classifying them as rather difficult to read.

## Discussion

### Choice of Support Tools and Principal Findings

Despite the availability of regularly updated clinical practice guidelines [5,6,9], studies report substantial variation in dentists’ knowledge regarding appropriate treatment approaches [13,14], which may, at least in part, be attributed to insufficient education on this topic during undergraduate training. It is likely that such knowledge gaps are increasingly being addressed through the use of digital tools, as they offer rapid access to relevant information and align with modern learning habits. Accordingly, the primary aim of this study was to compare different support tools in terms of their accuracy in clinical decision-making situations. Considering the upward trend of digital health technologies while still accounting for students’ preferences, we included 2 technologically advanced support tools (mobile app and chatbot) and a digital textbook representing a more traditional information source. To the best of our knowledge, this is the first study to compare different support tools specifically addressing emergency treatment questions related to dental trauma. Overall, the results indicated that the use of a support tool significantly increased accuracy compared with the control group, with no significant differences observed among the 3 sources. In recent years, the mobile health approach has gained increasing popularity, not only in daily clinical practice among health care professionals, but also as a means of delivering educational content [15,17]. In the field of dental traumatology, several mobile apps have been introduced. However, information regarding the dental trauma management according to the type of injury varied among the mobile apps [37]. Three of the apps were based on the widely accepted IADT guidelines, with the AcciDent app being the most popular and commonly used in Germany. Therefore, it was selected for inclusion in this study. In line with our results, previous studies have demonstrated that the use of a dental trauma app as a decision support tool significantly improves dental students` accuracy in answering multiple-choice questions related to the management of injured primary teeth, compared with a control group without access to informational resources [38,39].

Although this study indicated that the use of any of the evaluated support tools increases performance, the time required to get an answer differed significantly. Students using the mobile app and the chatbot completed the questionnaire within a mean time of 42.65 and 36.56 minutes, respectively, whereas those using the digital textbook required significantly more time (59.60 min). Moreover, it is important to note that the digital textbook included a keyword-based search function, which facilitates information retrieval. Consequently, it can be assumed that the use of a paper-based textbook would likely have resulted in even longer completion times.

### Item Quality

For the purpose of the study, students of the final academic year were asked to answer 25 dichotomous questions. The item quality analysis revealed an internal consistency (KR-20) of 0.59. While this value is below the commonly accepted threshold of 0.70 for high reliability, it may still be considered acceptable in the context of this exploratory study. The value likely reflects the intentional heterogeneity of the item content, which was designed to cover a broad range of clinically relevant topics regarding the emergency treatment of traumatic dental injuries. Since the primary aim was not to measure a single underlying construct, but rather to assess the accuracy of different support tools across a variety of question types, a lower internal consistency is expected and does not compromise the interpretability of the results. In terms of discrimination appropriateness, a difficulty analysis was conducted. While many items in the full dataset fell into the “easy” category (*P*_mean_=0.84, *D*_mean_=0.18), several still demonstrated substantial variation between groups, underscoring their discriminatory utility at the group level. To better reflect the discriminatory power of the items in relation to baseline student knowledge, difficulty, and discrimination indices were also calculated exclusively for the control group without support tool. Within this subgroup, the values were more balanced (*P*_mean_=0.77, *D*_mean_=0.20), suggesting that item performance was more aligned with typical student knowledge and less confounded by tool-specific effects. Moreover, since the purpose of the assessment was not to achieve maximum discrimination among individuals but rather to evaluate the accuracy of information derived from different sources, the inclusion of items reflecting clinically relevant knowledge, even when classified as “easy,” is justifiable.

### Performance of LLM-Based Support Tools

Recent advances in AI have accelerated the development of LLMs [21]. As these models continue to develop and become more accessible, their integration into health care settings has expanded considerably. Numerous studies have investigated LLMs as an assistive tool in medicine and dentistry regarding various domains, including treatment recommendations [34], emergency treatments [40], radiological and clinical diagnostics [41-43], education [24,44-46], and decision-making [47,48]. In the context of dental traumatology, several chatbots have been examined with regard to their accuracy in answering topic-specific questions [26,49-52]. These studies either simulated patient-initiated queries or potential questions posed by dental professionals. Moreover, methodological differences among these studies are evident in the type of question analyzed: patient-initiated questions were predominantly open-ended or case-related [49,50], while questions directed to professionals were assessed using multiple-choice or dichotomous formats [26,51,52]. With respect to the latter, reported accuracies for dichotomous questions varied widely (ranging from 10% to 80.81%) depending on the chatbot used (Google Bard, Google Gemini, Copilot F, Copilot P, ChatGPT-3.5, ChatGPT-4). In this study, ChatGPT-4o showed a mean accuracy of 86.40%. These discrepancies may also be explained by differences in the types of questions posed, as well as by continuous learning and training of the underlying models, which complicate direct comparisons between studies conducted at different time points.

### Role of Prompt Design

It has been demonstrated that he accuracy of recommendations by LLMs is largely contingent upon the input’s precision, correctness, and reasoning [24,34]. Various prompting strategies have been introduced and assessed to enhance the output response of LLMs [23,25,53,54]. These include prompting strategies like zero-shot, few-shot, or the thought-generation [23]. Further subtypes of prompting techniques have been described to enhance the precision and relevance of model responses. Role prompting, for example, instructs the model to adopt a specific perspective (such as responding to a clinical expert) in order to generate a domain-specific output. Chain-of-thought prompting encourages step-by-step reasoning to enhance the clarity of the response. Moreover, knowledge-guided prompting directs the model to rely on established guidelines or scientific sources, improving factual accuracy. Finally, the determination of the output format constrains the structure of the response, for example, by instructing the model to answer only with correct or false, or to respond in bullet points or tables [23,53]. Although prompt engineering is an emerging area of research, many users remain unaware of how the precision of a prompt can influence the model’s output [34]. A recent review on the innovation and application of LLMs in dentistry noted that even in LLM-related research, only a few studies have examined the impact of the input prompt design on the output quality [38,55,56]. In this study, the quality of the input prompt and the output response was assessed using Likert scales based on predefined criteria derived from the prompting strategies described above. Our results indicated that most students were not aware of the relevance of prompt formulation, resulting in a significant influence of the prompt quality on the output response. Even though the integration of AI-assisted tools in educational settings and, therefore, students’ familiarity with LLMs, may differ between countries, the importance of the input prompt can be considered universal. However, prompts should be locally adapted, since treatment guidelines or disease prevalence may vary across regions, which may otherwise lead to inappropriate treatment suggestions or implausible differential diagnoses.

### Role of Digital Tools in Learning

Previous research has demonstrated that a substantial proportion of medical and dental students rely on digital learning tools, frequently favoring them over conventional textbooks [57]. This tendency is especially pronounced in the context of short-term exam preparation, where efficiency and accessibility are key considerations [58,59]. Consequently, it is highly likely that students prefer to use apps or other digital tools to address knowledge gaps in clinical situations rather than relying on traditional textbooks, particularly when immediate support is required.

Although the groups using ChatGPT-4o and the AcciDent app performed equally in terms of accuracy, it is worth noting that the content provided by a mobile app can be controlled by its developers, for example, by aligning it with international guidelines, which increases its reliability and safety. In contrast to mobile apps, which often require regular updates and may involve purchase costs, one advantage of the use of LLM-based chatbots is the continuous access to a broad and dynamically evolving source of information. Nevertheless, educators and students should be encouraged to critically evaluate the AI-generated feedback in order to develop their clinical reasoning and problem-solving skills, rather than relying unreflectively on the model’s output. Excessive dependence on AI may otherwise impair the development of independent clinical judgment and practical skills [60]. Therefore, conventional textbooks remain essential, as they provide reliable fundamental knowledge to understand and internalize clinical concepts over the long term, and help to mitigate the risks and limitations associated with LLMs.

Moreover, adequate application of LLMs includes a precise prompt design, as this directly influences the clarity and accuracy of AI-generated responses. In medical and dental education, this ability can be regarded as a core component of LLM literacy, which is teachable and measurable [61,62]. Aligning this competency with established frameworks, such as digital health literacy or AI-related competencies in health professions education, reinforces its curricular and theoretical relevance [61-63]. Ultimately, an acquired, ideally curriculum-integrated, competence in using LLMs enables students and professionals to engage with these tools in a safe, informed, and reflective manner.

### Strengths and Limitations

In general, the format of the question has a significant impact on chatbot performance [51]. In this study, the dichotomous format was chosen for its simplicity, ease of automated scoring, and efficiency in covering a broad content range within a limited timeframe. It also minimized the influence of distractor quality, which can significantly affect the validity of multiple-choice items. Poorly constructed distractors—such as implausible or irrelevant answer choices—can reduce item discrimination and compromise test reliability, particularly in AI-assisted educational settings [64,65]. However, the use of dichotomous questions is not without limitations. Prior research has shown that the performance of LLMs can vary significantly depending on the question type, with multiple-choice formats potentially offering a more nuanced reflection of clinical reasoning processes [66,67]. Moreover, true/false formats have a higher chance of guessing (50%), with limited possibility to reflect complex clinical scenarios, and a reduced ability to discriminate between different levels of understanding [68]. Future studies may benefit from incorporating multiple-choice or open-ended formats as well as clinical vignettes to better evaluate critical thinking and complex decision-making skills, especially in the context of AI-assisted learning [69,70].

Regarding more complex question formats, different approaches have been explored. For instance, ChatGPT-3.5 was shown to respond adequately to patient-related advice-seeking questions across medical scenarios, although without providing appropriate or personalized advice [71]. In internal medicine, another study assessed the diagnostic accuracy and the ability of stating differential diagnoses of ChatGPT-3.5 and ChatGPT-4 using case vignettes. The authors highlighted the potential utility of LLMs as a supplementary tool [72]. This finding was supported by a similar study on orofacial pain [73]. However, although GPT-4 can augment diagnostic workflows, particularly in primary care or educational settings, it does not yet outperform clinical experts [73]. With regard to the impact of LLM use on physicians’ diagnostic reasoning when working with clinical vignettes in internal, family, or emergency medicine, it was demonstrated that providing physicians with an LLM as a diagnostic aid did not significantly improve clinical reasoning compared with conventional resources [74]. Interestingly, however, the LLM alone performed significantly better when given standardized zero-shot prompts than when used as a supportive tool by physicians, highlighting the importance of input prompt quality [74]. As mentioned before, the results of this study are limited to a question format with a relatively high likelihood of guessing. Even within this “easier-to-answer” format, however, we were able to demonstrate the impact of prompt formulation. It is reasonable to assume that in more complex clinical case scenarios, the quality of the input prompt will become even more critical.

This study involved a relatively small sample of 59 final-year dental students, a limitation primarily dictated by the cohort size of the final academic year. While the sample size constrains generalizability to some extent, the decision to restrict participation to senior students was intentional. This group was presumed to represent students with adequate clinical knowledge to comprehend the clinical implications of traumatic dental injuries, despite not having formal education on the topic. Including only students at the end of their undergraduate education ensured a more uniform baseline of dental knowledge and minimized potential bias due to major curricular differences across earlier academic years. Although our results are limited to the included cohort, it can be assumed that all evaluated support tools are helpful in trauma-related questions or clinical situations. However, users with less clinical experience and prior knowledge may face a greater risk of relying on incorrect chatbot responses. For these groups in particular, precise formulation of input prompts and critical appraisal of AI-generated output will be especially important. Our results demonstrated that any of the support tools increased the students’ performance; however, there were differences regarding the total time required. It can be assumed that users who were more familiar with a specific tool required less time, as its handling was easier and more intuitive for them. Students’ familiarity with the tools was not assessed prior to the study, which should be considered a limitation. However, participants were randomly allocated to the groups to minimize this potential influence.

One of the strengths of the study is that the students’ input entries to the chatbot were considered and evaluated. Using clearly defined criteria, we achieved a high interrater agreement (Cohen κ) for both the input and the output quality, demonstrating the straightforward and reproducible application of the predefined Likert scale. Unlike previous studies that assessed chatbot accuracy and consistency using identical prompts, this study accounted for both the interindividual variability in question formulation and decision-making based on chatbot recommendations, a factor often disregarded in earlier evaluations [26,51,52].

### Conclusions

The present findings indicate that all evaluated support tools improved the students’ performance when answering dental trauma questions. Both ChatGPT-4o and the AcciDent app demonstrated high accuracy while requiring less time than the digital textbook group. However, the performance of ChatGPT-4o depends on the precision and specificity of the input prompt, an aspect students should be made aware of when using it as an information source. In general, AI-based apps have the potential to transform dental education by enhancing learning experiences and supporting instructional practices. At the same time, educators and students should be encouraged to critically evaluate the AI-generated feedback in order to address the current risks and limitations associated with the use of LLMs.

## Appendix

Multimedia Appendix 1 Authors’ translation of the used questionnaire, which was originally distributed in German.

Multimedia Appendix 2 Detailed item analysis.

Multimedia Appendix 3 CONSORT-eHEALTH checklist (V 1.6.1).

## Abbreviations

AI: artificial intelligence
GPT: generative pretrained transformer
IADT: International Association of Dental Traumatology
KR-20: Kuder Richardson formula 20
LLM: large language model

## Data Availability

The datasets generated or analyzed during this study are available from the corresponding author on reasonable request.
