# Supplementary material for: Evaluating ChatGPT-4o as an Educational Support Tool for the Emergency Management of Dental Trauma: Randomized Controlled Study Among Students
Source: JMIR Med Educ. 2025 Nov 20;11:e80576. doi: 10.2196/80576 (PMC12679074; doi:10.2196/80576)
Supplement: Multimedia Appendix 1 [file mededu_v11i1e80576_app1.docx]

**Appendix 1**

**Table S1.** Authors’ translation of the used questionnaire, which was originally distributed in German. Overview of the 25 dichotomous items used, indicating the statements that participants were asked to classify as true or false.

| 1 | In cases of enamel-dentin fractures with pulp exposure, immediate endodontic access is indicated. |
| --- | --- |
| 2 | In cases of an enamel-dentin fracture without pulp exposure, adhesive reattachment of the fractured tooth fragment is a possible treatment option. |
| 3 | A fiberglass splint is particularly suitable for splinting displaced teeth. |
| 4 | Replantation of an avulsed primary tooth is recommended if the injury occurred within the past 15 minutes. |
| 5 | In cases of anamnestical and clinical suspicion of dental trauma, three-dimensional imaging diagnostics should generally be performed. |
| 6 | Crown fractures involving enamel only must be sealed within the first 5 days. |
| 7 | An avulsed permanent tooth should be stored in saline solution as soon as possible to enable successful replantation. |
| 8 | In crown fractures without pulp involvement, the exposed dentin should be sealed immediately. |
| 9 | In the case of an ideally stored permanent tooth with completed root development, splinting is necessary after replantation. |
| 10 | In cases of a root fracture (intra-alveolar fracture), splinting should be performed after repositioning for at least 4 weeks. |
| 11 | As part of the initial treatment following dental trauma, the patient’s polio vaccination status should be reviewed. |
| 12 | In cases of enamel-dentin fractures with pulp exposure, immediate endodontic access is indicated. |
| 13 | In cases of tooth luxation, a rigid splint should be applied after repositioning. |
| 14 | In cases of a concussion, the affected tooth presents no increased mobility. |
| 15 | In luxated teeth with a displacement of more than 2 mm and fully developed roots, endodontic treatment should be initiated during the splinting phase. |
| 16 | In cases of tooth extrusion exceeding 2 mm, extraction should be considered for teeth with completed root development. |
| 17 | Root canal treatment of replanted avulsed teeth with completed root development should be initiated within 10 days after trauma. |
| 18 | As part of the initial treatment, patients with enamel-dentin fractures involving the pulp should receive systemic antibiotic therapy. |
| 19 | If a patient presents after an accident with severe headache and nausea, referral to the nearest hospital to rule out a traumatic brain injury is advised prior to initiating dental treatment. |
| 20 | In cases of root fracture, the duration of splinting depends significantly on the fracture level (cervical vs. apical). |
| 21 | If the alveolar process is fractured without any luxation of the teeth in the affected area, root canal treatment should be initiated as soon as possible. |
| 22 | In the presence of positive percussion sensitivity on the day of the accident, root canal treatment should be initiated as soon as possible. |
| 23 | In cases of intrusion, the patient should be informed that root resorption is highly likely to occur. |
| 24 | In cases of lateral luxation, an accompanying alveolar process fracture should always be considered. |
| 25 | In the case of minor intrusion of a central incisor in a 7-year-old patient, spontaneous reeruption should be awaited. |
