# Supplementary material for: Evaluating ChatGPT-4o as an Educational Support Tool for the Emergency Management of Dental Trauma: Randomized Controlled Study Among Students
Source: JMIR Med Educ. 2025 Nov 20;11:e80576. doi: 10.2196/80576 (PMC12679074; doi:10.2196/80576)
Supplement: Multimedia Appendix 2 [file mededu_v11i1e80576_app2.docx]

**Appendix 2**

**Table S2.** Difficulty (P) and discrimination (D) indices for all 25 test items. Values are presented for the overall sample as well as separately for the control group.

| Item | P_Total_ | P_Control_ | D_Total_ | D_Control_ |
| --- | --- | --- | --- | --- |
| 1 | 0.76 | 0.93 | 0.23 | 0.00 |
| 2 | 0.97 | 0.93 | 0.06 | 0.33 |
| 3 | 0.58 | 0.36 | 0.15 | 0.07 |
| 4 | 0.81 | 0.36 | 0.35 | 0.4 |
| 5 | 0.95 | 0.86 | 0.12 | 0.13 |
| 6 | 0.98 | 1.00 | 0.00 | 0.00 |
| 7 | 0.58 | 0.29 | 0.45 | 0.60 |
| 8 | 1.00 | 1.00 | 0.00 | 0.00 |
| 9 | 0.95 | 0.86 | -0.01 | -0.20 |
| 10 | 0.78 | 0.57 | 0.22 | 0.47 |
| 11 | 0.83 | 0.79 | 0.35 | 0.33 |
| 12 | 1.00 | 1.00 | 0.00 | 0.00 |
| 13 | 0.78 | 0.71 | 0.47 | 0.13 |
| 14 | 0.83 | 0.43 | 0.47 | 0.80 |
| 15 | 0.49 | 0.36 | 0.56 | 0.07 |
| 16 | 0.97 | 1.00 | 0.06 | 0.00 |
| 17 | 0.47 | 0.64 | 0.11 | 1.00 |
| 18 | 0.97 | 0.93 | 0.12 | 0.33 |
| 19 | 0.98 | 1.00 | 0.00 | 0.00 |
| 20 | 0.73 | 0.50 | 0.28 | 0.27 |
| 21 | 0.98 | 0.93 | 0.06 | 0.00 |
| 22 | 0.98 | 1.00 | 0.00 | 0.00 |
| 23 | 0.92 | 0.86 | 0.24 | 0.00 |
| 24 | 0.83 | 0.86 | 0.23 | 0.33 |
| 25 | 0.97 | 1.00 | 0.06 | 0.00 |
